# The Cancer Research Database (CRDB): Integrated Platform to Gain Statistical Insight Into the Correlation Between Cancer and COVID-19

**DOI:** 10.2196/35020

**Published:** 2022-06-10

**Authors:** Shahid Ullah, Farhan Ullah, Wajeeha Rahman, Dimitrios A Karras, Anees Ullah, Gulzar Ahmad, Muhammad Ijaz, Tianshun Gao

**Affiliations:** 1 S-Khan Lab Mardan Khyber Pakhtunkhwa Pakistan; 2 Department General Faculty of Science National and Kapodistrian University of Athens Athens Greece; 3 Kyrgyz State Medical University Bishkek Kyrgyzstan; 4 Research Center The Seventh Affiliated Hospital of Sun Yat-sen University Shenzhen China

**Keywords:** cancer database, COVID-19, CRDB, genomics, PHP, CRDB, cancer and COVID-19, cancer statistics, cancer research, health database, research platform

## Abstract

**Background:**

The advancement of cancer research has been facilitated through freely available cancer literature, databases, and tools. The age of genomics and big data has given rise to the need for cooperation and data sharing in order to make efficient use of this new information in the COVID-19 pandemic. Although there are many databases for cancer research, their access is not easy owing to different ways of processing and managing the data. There is an absence of a unified platform to manage all of them in a transparent and more comprehensible way.

**Objective:**

In this study, an improved integrated cancer research database and platform is provided to facilitate a deeper statistical insight into the correlation between cancer and the COVID-19 pandemic, unifying the collection of almost all previous published cancer databases and defining a model web database for cancer research, and scoring databases on the basis of the variety types of cancer, sample size, completeness of omics results, and user interface.

**Methods:**

Databases examined and integrated include the Data Portal database, Genomic database, Proteomic database, Expression database, Gene database, and Mutation database; and it is expected that this launch will sort, save, advance the understanding and encourage the use of these resources in the cancer research environment.

**Results:**

To make it easy to search valuable information, 85 cancer databases are provided in the form of a table, and a database of databases named the Cancer Research Database (CRDB) has been built and presented herein. Furthermore, the CRDB has been herein equipped with unique navigation tools in order to be explored by three methods; that is, any single database can be browsed by typing the name in the given search bar, while all categories can be browsed by clicking on the name of the category or image expression icon, thus serving as a facility that could provide all the category databases on a single click.

**Conclusions:**

The computational platform (PHP, HTML, CSS, and MySQL) used to build CRDB for the cancer scientific community can be freely investigated and browsed on the internet and is planned to be updated in a timely manner. In addition, based on the proposed platform, the status and diagnoses statistics of cancer during the COVID-19 pandemic have been thoroughly investigated herein using CRDB, thus providing an easy-to-manage, understandable framework that mines knowledge for future researchers.

## Introduction

Cancer is a category of diseases causing irregular cell growth with the ability to infiltrate or spread to other areas of the body. As of 2019, approximately 18 million new cases are reported per year [1], among which 22% of cancer deaths are caused by tobacco use and 10% are caused by obesity, an unhealthy diet, a lack of physical activities, or excessive alcohol use [2]. In the past 2 or 3 decades, recent data have shown that approximately 5%-10% of cancers are caused by genetic disorders [3]. Patients with cancer seem to exhibit exacerbated conditions and a higher mortality rate when exposed to the virus [4]. The COVID-19 pandemic has spread over the world. As in 18 October 2021, there have been 219 million confirmed cases and 4.55 million deaths worldwide, with the number of cases continuing to rise in 216 countries [5]. Patients with cancer are thought to be particularly prone to SARS-CoV-2 infection and the development of more severe COVID-19 symptoms, which could be related to a systemic immunosuppressive condition caused directly by tumor growth and indirectly by anticancer therapy’s side effects [6]. Infection can affect people of various ages, but in most situations, disease severity is linked to age limit and pre-existing disorders that decrease immunity, such as cancer. COVID-19 has been linked to an increased risk of severe sickness and death among patients with cancer, according to several studies [7]. Brunello et al [8] suggested that people with cancer are faced with two challenges that potentially lead to death: one is from getting cancer and the other is COVID-19—the latter resulting from undertreatment or overtreatment conditions. Initial investigations revealed that patients with cancer were more likely to contract the virus and become infected with COVID-19.

Because of the effects of antineoplastic therapy, supportive drugs including steroids, and the immunosuppressive qualities of cancer, people with cancer could be immunocompromised [5]. The advancement of modern genomic technology—such as microarrays, proteomics, transcriptomics, and gene sequencing—and the serious situation resulting from the COVID-19 pandemic have resulted in the generation of a huge amount of data [9,10]; therefore, the first challenge of these multi-omics cancer and COVID-19 data was the design and usage of electronic databases to store and manage the large amount of knowledge [11]. A number of databases have been published in this research area, which have explained the wide-ranging information about cancer research and COVID-19 [10,12-16], such as The Cancer Genome Atlas (TCGA), RespCanDB, cBioPortal, Co-19PDB, and ICGC, these databases have been exploring, analyzing, and visualizing multidisciplinary genomics data. Further, we have presented a comparison table with previously published work, in which we have noticed a considerable growth in the number of databases, and we have provided the list of all the cancer databases and have built a database of the databases named the Cancer Research Database (CRDB), with the improvement highlighted in Table 1. To make it easier for researchers and the scientific community, a well-organized and easily accessible platform is required, where all cancer research data can be accessed with a single click. To that end, we have gathered almost all cancer databases and classified them into six categories based on data types: Data Portal database, Genomic database, Proteomic database, Expression database, Gene database, and Mutation database; this would provide an easy way to search data, and users can directly type the name of the needed databases in the search bar or can click the required category, which will lead them to all the databases with a single click. In addition, we have obtained deep insight into the link between cancer and the COVID-19 pandemic, having explained up and down of cancer, new cases, death ratios, etc, before and during the COVID-19 pandemic.

**Table 1:** Comparison of the Cancer Research Database with other published work.

Database	Databases, n	Type	Year	Component	Journal	Reference
Cancer research database	98	Database+list	2022	Cancer	N/A^a^	N/A
Munich Information Center for Protein Sequences	22	List	2011	Different categories	*Nucleic Acids Research*	[17]
No name	6	Database	2018	Aging	*Nucleic Acids Research*	[18]
COVID-19 pandemic database	59	Database+list	2021	COVID-19	*Computer Methods and Programs in Biomedicine Update*	[10]
Swiss Institute of Bioinformatics	12	Database	2016	Different categories	*Nucleic Acids Research*	[19]
Human cancer databases	58	List	2015	Cancer	*Oncology Reports*	[20]
No name	38	List	2014	Hepatology	*Journal of Hepatology*	[21]
LiverAtlas	53	Databases	2013	Liver	*Liver International*	[22]
No name	16	List	2015	Cancer	*Genomics, Proteomics & Bioinformatics*	[3]

^a^N/A: not applicable.

Previously, we have published several articles in well-known journals, such as the database of Phospho-sites in Animals and Fungi [23] in *Scientific Reports*, the Circadian Gene Database [24] in *Nucleic Acids Research*, Co-19PDB [10] in *Computer Methods and Programs in Biomedicine Update*, DataBases relevant to Human Research [25] in *Future Science* and DataBase of Plant Research [26]; we have provided 15 databases to the scientific community during the COVID-19 pandemic, which can be accessed on the internet [27].

## Methods

### Construction of the CRDB and Content

**Figure 1:**
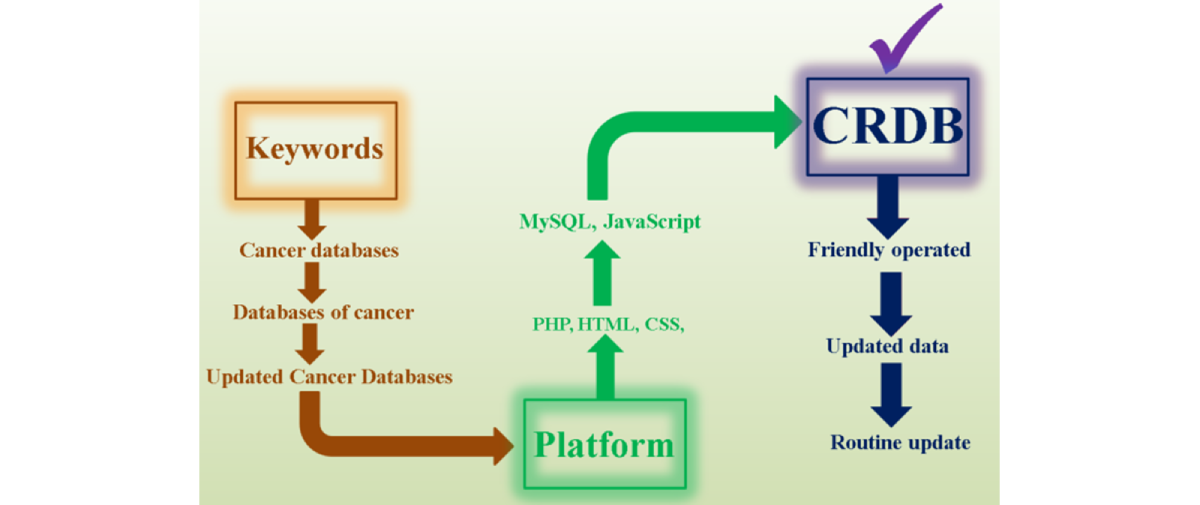
Flowchart and procedure for the collection and integration of cancer databases and the construction of the Cancer Research Database (CRDB).

We integrated the data from multiple different sources including PubMed, Google, Google Scholar, etc. We used various keywords such as “Cancer database,” “cancer database list,” and “database of cancer” as search terms to retrieve published cancer-related databases with the help of PubMed. To circumvent missing data, we have manually collected the latest cancer databases from *Nucleic Acids Research*, and *Genomics, Proteomics & Bioinformatics*, which are the leading journals on the database issue. We only collected all cancer databases and have removed all nonfunctional links and programming platforms such as PHP, MySQL, HTML, CSS, and JavaScript have been used to construct the CRDB. Figure 1 shows all the procedures of our database. Finally, we provided a compressive cancer research database to the scientific community, which is easy to operate and will be updated over time.

### Database Classification

Several articles have been published in this research area [28-30], each has its their own classification of databases based on its function, application, technological feature, and organism, such as human and mouse [29], Plant [26], Drosophila [31], fungi, COVID-19, etc. According to such published works, we have also classified the cancer databases into six categories: Data Portal database, Genomic database, Proteomic database, Expression database, Gene database, and Mutation database—their details are given bellow.

#### Expression Databases

**Figure 2:**
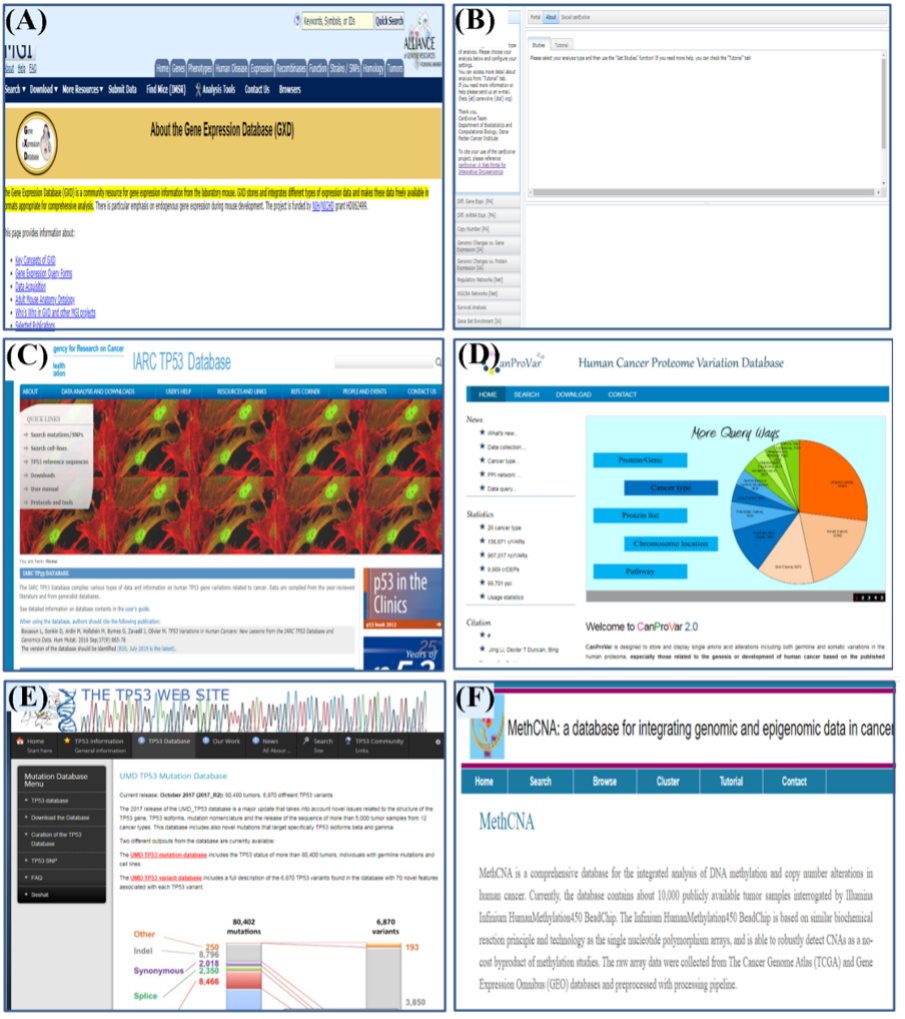
Main pages of some commonly using cancer databases. (A) A screenshot of the Expression Database named GXD, (B) a screenshot of the Data Portal category named CNVs (copy number variations), (C) “IARCTP53”: a database of the Gene category, (D, E, and F) main pages of Proteomic database, Mutation database, and Genomic database respectively.

In cancer expression databases, the expression levels of thousands of genes can be continuously measured under particular experimental environments and conditions resulting from marked advancements in DNA microarray technology. This technology made it possible to understand life at the molecular level, and enables us to generate large-scale gene expression data. It has also been applied in a wide range of applications such as cancer prediction, diagnosis, and drug discovery, which are very important issues for cancer treatment [32]. Some well-known expression databases including BioXpress [33], miRCancer [34], and Gene Expression Database [35] are shown in Figure 2A.

#### Data Portal Databases

Data Portal is a type of database that provides comprehensive genomic, epigenomic, transcriptomic, and proteomic data. a large number of data are publicly available for anyone in the research community and are used to diagnose, treat, and prevent cancer [12]. There are different published databases such as The European Genome-phenome Archive (EGA), which is a data center for all types of sequencing and genotyping experiments. Almost 58% of all studies in the EGA are related to cancer [36]. The “CanEvolve” database fulfills the need for data integration and interpretation. It contains data from 90 studies involving more than 10,000 patients. Data analysis can be performed at different levels: primary analysis including mRNA, microRNA (miRNA), and protein expression, genome variations, and protein–protein interactions; integrative analysis of gene and miRNA expression, gene expression, and copy number variations, and gene set enrichment analysis; network analysis; and survival analysis [37]; the main page of this database is shown in Figure 2B.

#### Gene Databases

Gene databases collect various types of gene data and information related to cancer [38] and also provide help to researchers in understanding the genetic architecture of complex diseases and improve the accuracy of diagnosis and the effectiveness of therapy [39]. Various databases have been published such as the “IARCTP53” shown in Figure 2C; this database compiles various types of data and information on human *TP53* variations related to cancer [38]. The “TGDBs” gene database includes mechanisms of oncogenic activation, regulation, frequency of involvement in various tumor types, and chromosomal location. Data about the encoded proteins includes the cell type in which they are found, subcellular location, DNA-, protein-, and ligand-binding, role in development, and normal biochemical function [40].

#### Proteomic Databases

Cancer proteome databases encompass tumor tissues, cells, and biological fluids to interpret signaling pathways, identify signatures related to tumor initiation, invasion, and metastasis, and determine analytical, predictive, and prognostic markers [41], and also help determine the molecular details of proteome differentiation in various human tissues and organs, thus greatly improving our understanding of disease and human biology [23,42]. A number of databases have been published in this research area such as those shown in Figure 2D: “CanProVar” is designed to store and display single amino acid alterations including both germline and somatic variations in the human proteome, particularly those related to the genesis or development of human cancer based on the published studies and sources [43].

#### Mutation Databases

Mutation databases play an important role in science, diagnostics, and genetic health care and can play a vital role in life and death decisions. These databases are extensively used, but only gene- or locus-specific databases have been previously reviewed for their utility, accuracy, completeness, and currency [44]. Mutations in the tumor suppressor gene *TP53* are associated with a variety of cancers [45]. More than 50% of the human tumors harbor *TP53* mutations, resulting in a collection of over 45,000 somatic and germline mutations in the UMDTP53 database, as shown in Figure 2E. Analyses of these mutations have been helpful for improving our knowledge on the structure-function relations within the TP53 protein [46]. A number of databases have been published in this research area, which are of marked utility in the scientific community.

#### Genomic Databases

The Cancer Genome Database represents one of numerous international groups dedicated to performing wide-ranging genomic and epigenomic studies of selected cancer types to develop our understanding of disease and provide an open-access resource for international cancer research [47]. This database is aimed at improving the understanding of the molecular basis of cancer development [48]. Several databases have been published in this research area; for example, the MethCNA comprehensive database for genomic data in human cancer (Figure 2F). Per a most recent publication, this database contains approximately 10,000 tumor samples covering 37 cancer types. All the data were collected from the TCGA and the National Center for Biotechnology Information and were evaluated using a pipeline that combined multiple computational resources and tools [49]. BioMuta is a single-nucleotide variation and disease association database where variations are mapped to genomes and RefSeq nucleotide entries and are incorporated through UniProtKB/Swiss-Prot positional coordinates. The recent version of BioMuta contains only nonsynonymous single-nucleotide variations associated with cancer [50].

## Results and Discussion

### Database Statistics

**Figure 3:**
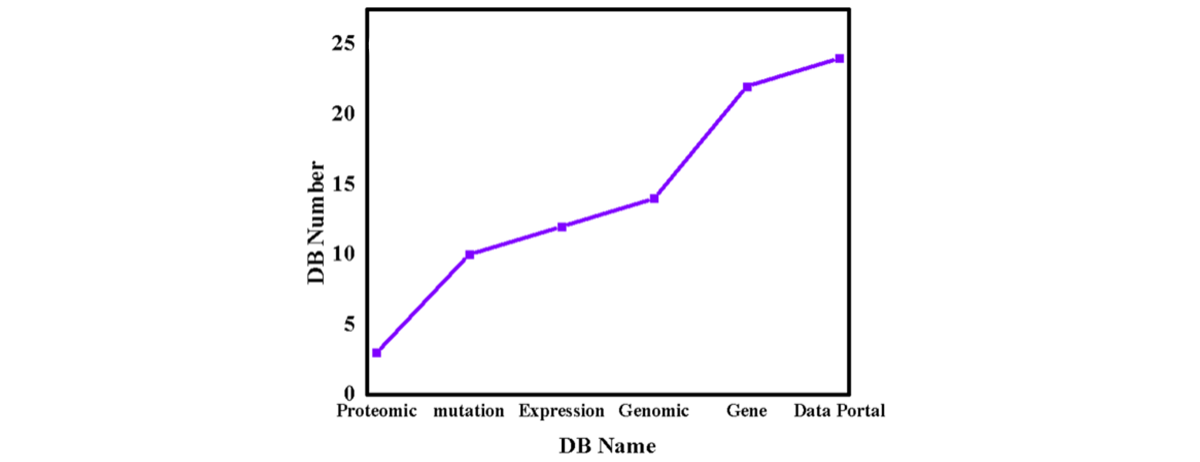
The statistics data of the Cancer Research Database (DB)—distribution of the database category.

In this work, we have provided almost all cancer databases (Table S1 in Multimedia Appendix 1) and shown the year-wise growth of the CRDB. Figure 3 shows the magnitude of category-wise growth of the databases. Table 2 shows the year-wise growth distribution of cancer databases, which marks tremendous growth and is an achievement for the cancer scientific community. Further, we have modified or deleted all the nonfunctional and inaccessible database links and have provided a new, updated cancer database in the form of a database named CRDB and a table (Table S1 in Multimedia Appendix 1).

**Table 2:** Year-wise growth of the Cancer Research Database.

Year	Database growth, %
1999	1
2004	1
2005	1
2006	2
2007	2
2008	2
2009	2
2010	5
2011	6
2012	2
2013	7
2014	5
2015	6
2016	6
2017	11
2018	7
2019	7
2020	14
2021	12

### Usage of the CRDB

**Figure 4:**
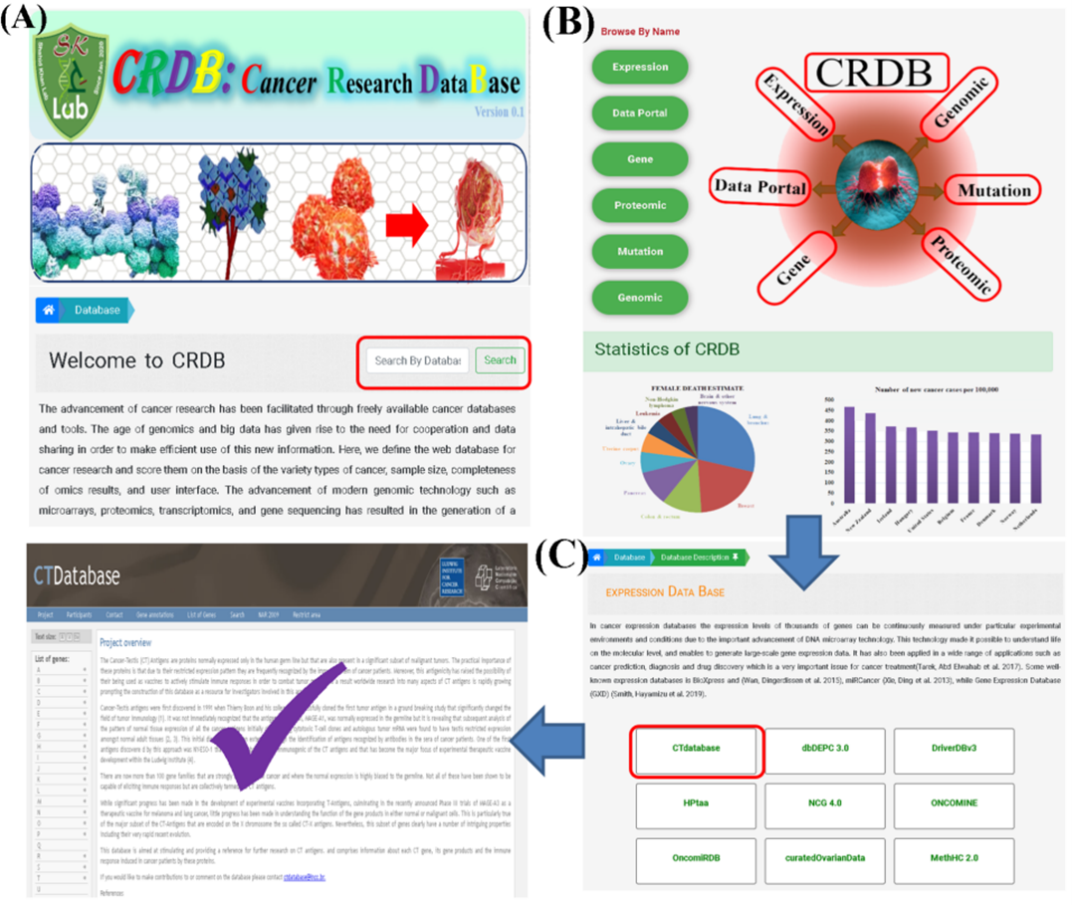
Browse options of the Cancer Research Database (CRDB). (A) Can be browsed by typing the name. (B) Can be browsed by category name or image expression. (C) An example and the final result.

The CRDB has been developed to provide an easy and user-friendly search experience; for easier and faster search, three options are provided for finding cancer databases. First, browsing can be carried out by typing the name of the database in the search bar, which is highlighted in Figure 4A, or by clicking on the name of the category or image expression, which is shown in Figure 4B. with CRDB statistics as well, which will lead to the category list page (Figure 4C), and a brief overview with the original link of the required search will be accessed by clicking the needed database. Further, for a specific database search, the “CT Database” is used as an example from the expression Databases to make it more user-friendly.

### Example of Cancer Diagnosis During the COVID-19 Pandemic

As of the previously reported reductions in cancer screening and other preventive care visits during the COVID-19 pandemic, the number of new cancer cases in 2020 is likely to be smaller than anticipated. According to one survey of diagnostic results, there was a 46% decrease in diagnosis of six different cancers (colorectal, pancreatic breast, lung, esophageal, and stomach cancer) from March 1 to April 18, 2020, relative to the period between January 6, 2019, and February 29, 2020, varying from a 25% decrease in the detection rate of pancreatic cancer to a 52% decrease in that of breast cancer [51,52]. Another study found that new CRC diagnoses were 30% lower from January to mid-April 2020 relative to the same timeframe in 2019 [53]. Across the world, similar losses have been noted, including those in the United Kingdom [54], the United States [54], and the Netherlands [55]. While these preliminary observations may provide insight into the pandemic’s effect on cancer diagnosis, population-based cancer registry evidence and the degree to which these delays may lead to more advanced-stage disease will not be available for some time.

### COVID-19 and Cancer

**Figure 5:**
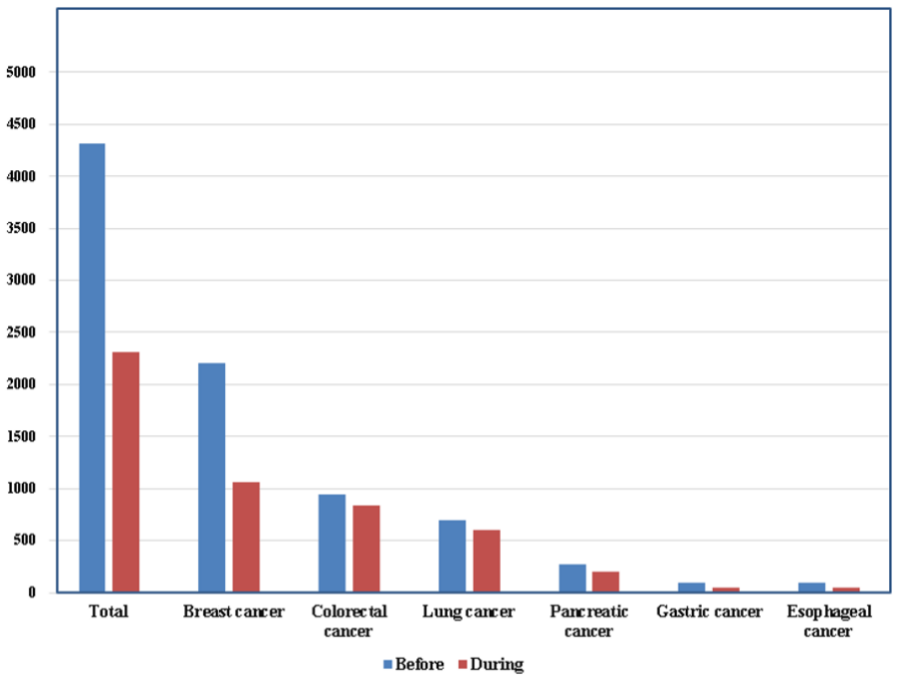
The number of cancers detected before and during the COVID-19 pandemic.

People with active cancer are more vulnerable to infectious pathogens as a result of a compromised immune system due to the malignancy and its treatment (eg, surgery and chemotherapy). This has raised fears that COVID-19–related problems and mortality may be more common among patients with cancer [56]. According to a 2020 study, patients with cancer may be at a higher risk of COVID-19 than those without cancer [57]. COVID-19 infection can impact persons with a wide range of hematologic diseases; however, the risk of infection is lower in patients with chronic myeloid proliferative neoplasms such as chronic myeloid leukemia and greater in persons on immunosuppressive medication [57,58]. A study at a tertiary care hospital in Wuhan, China, reported that patients with lung cancer above the age 60 years are at a high risk of COVID-19 locally [14,58,59] and worldwide [59,60]. It was also revealed that of the many cancer types, people with lung cancer who are over 60 years old are especially susceptible to COVID-19. Although it may seem intuitive that people with a defective respiratory epithelium are more susceptible to rapid virus entry into the lungs [61]. Another study shows a decrease in 6 types of cancer, with the total number of detected cancers being 4310 before and 2310 during the COVID-19 pandemic, with breast cancer ranking the highest with 2208 cases before and 2310 cases during the COVID-19 pandemic, followed by colorectal cancer at 946 cases before and 840 cases during the pandemic, and Figure 5 shows details regarding all 6 cancer databases before and during the COVID-19 pandemic [52].

### Global Cancer Rate

**Figure 6:**
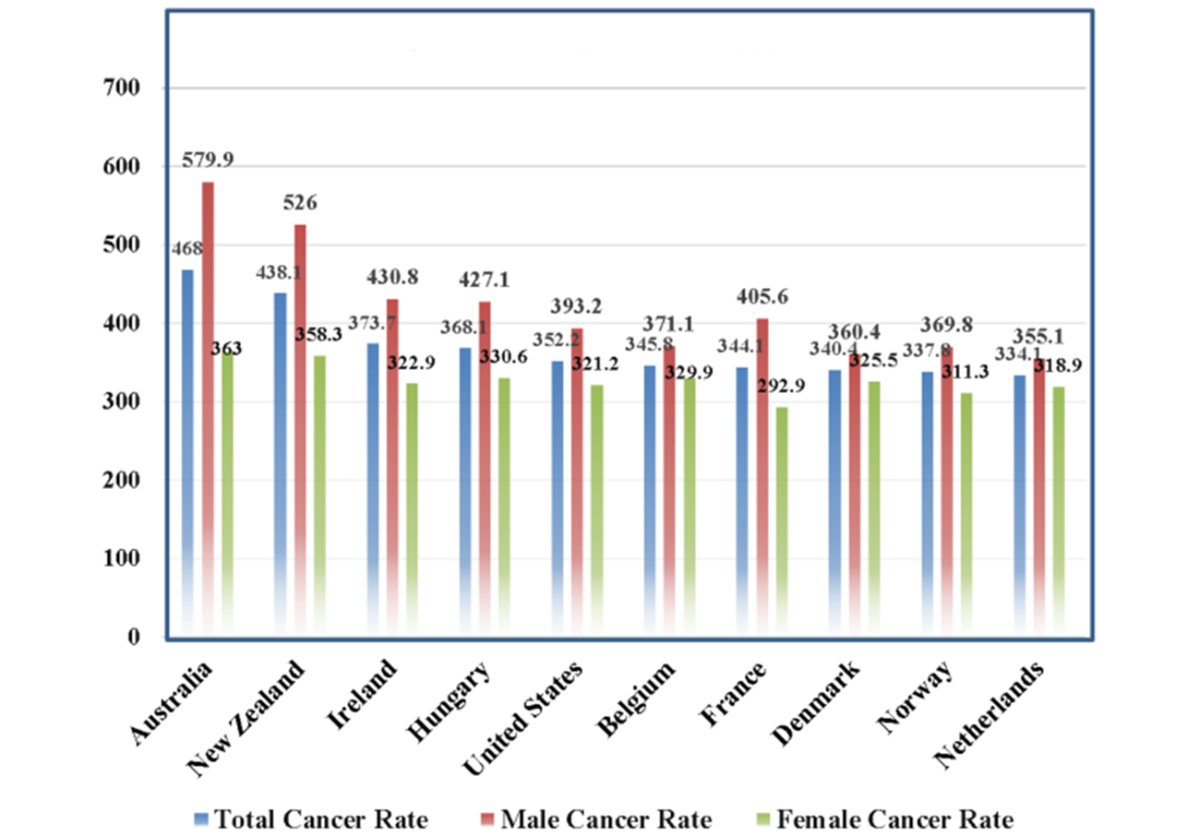
Country-wise cancer rate with an age-standardized average (per 100,000 population).

According to American institute for cancer research [62], the rates of all cancers have increased in almost in all countries, the privation of which is the major task for public health in 2021. Decreasing the cancer rate involves coordinated and comprehensive intervention from all facets of society, particularly the public sphere, civil society, and health and other occupations. Figure 6 shows the top 10 country-wise cancer rate from Oceania, Europe, and North America, in which the highest rate of cancer was reported in Australia (468.0 people per 100,000 population). For these 10 countries, the age-standardized average was at least 320 people per 100,000 population. With 579.9 men per 100,000 population, the age-standardized average was at least 360 people per 100,000 population, and with 363.0 women per 100,000 population, the age-standardized average was at least 300 people per 100,000 population.

### New Cases and Deaths

The American Cancer Society Cancer Action NetworkSM works worldwide to increase the quality of care for patients with and survivors of cancer. With time and the emergence of new cases worldwide, we have compiled a list of the top 10 cancers diagnosed in the United States in 2021. Table 3 shows the number of new cancer cases, with breast cancer in women and prostate cancer in men ranking first (30% cases) and second (26%), respectively. Although mortality estimates are shown in Table 3, lung and bronchial cancer showed the same rates in both male and female patients and ranked the highest, followed by breast and prostate cancer.

**Table 3:** Rates of new cancers and mortality between male and female patients.

Cancer type	New cancers, %			Mortality, %	
	Male	Female	Male		Female
Prostate	26	N/A^a^	11		N/A
Breast	N/A	30	N/A		15
Lung and bronchial	12	13	22		22
Colorectal	8	8	9		8
Urinary bladder	7	—^b^	4		—
Skin melanoma	6	5	—		—
Kidney and renal pelvis	5	3	—		—
Non-Hodgkin lymphoma	5	4	4		3
Oral cavity and pharynx	4	—	—		—
Leukemia	4	3	4		3
Pancreatic	3	3	8		8
Uterine corpus	N/A	7	N/A		4
Brain and other nervous system regions	—	—	3		3
Liver and intrahepatic bile duct	—	—	6		3
Esophageal	—	—	4		—
Ovarian	N/A	—	N/A		5

^a^N/A: not applicable.

^b^—: not determined.

### Conclusions

A biological database provides facilities for storing, organizing, and retrieving biological data such as DNA, RNA, carbohydrates, proteins, and cancers. It can be easily viewed, managed, and modified. A number of papers have been published in this research field, which have their own classification of cancer databases based on their function, use, certain technical aspects, and on species such as human, mouse, plant, and fungi. According to such published studies, we have classified the cancer databases into six categories: Data Portal database, Genomic database, Proteomic database, Expression database, Gene database, and Mutation database. Further, we have collected almost all cancer databases with a short introduction and have updated or removed all nonfunctional links. Furthermore, we have understood the current situation of cancer and its correlation with COVID-19; for example, the up-down, mortality, and new case count based on continent and countries, etc. Our database can be searched through an easy-to-use, user-friendly method, can be searched by clicking on category name of image expression, or users can type the name of needed databases in the given search bar and they will be updated with time. In addition, we have examined the status and diagnoses of cancer during the COVID-19 pandemic and have provided easy and understandable information for future researchers.

## Appendix

Multimedia Appendix 1 Cancer databases.

## Abbreviations

CNV: copy number variation
CRDB: cancer research database
EGA: European Genome-phenome Archive
miRNA: microRNA
TCGA: The Cancer Genome Atlas

## Availability of Data and Material

These data will be available under the journal rule and regulation. To avoid future conflict and plagiarism issue, CRDB database is uploaded on the internet [63] so that we have provided some content in this article.
