# Supplementary material for: The Cancer Research Database (CRDB): Integrated Platform to Gain Statistical Insight Into the Correlation Between Cancer and COVID-19
Source: JMIR Cancer. 2022 Jun 10;8(2):e35020. doi: 10.2196/35020 (PMC9191331; doi:10.2196/35020)
Supplement: Multimedia Appendix 1 [file cancer_v8i2e35020_app1.docx]

| **Category** | **DB-Name** | **DB-Link** |
| --- | --- | --- |
| Data Portal | CPRG | <http://www.broadinstitute.org/software/cprg> |
|  | cBioPortal | <http://www.cbioportal.org/public-portal/> |
|  | canSAR | <https://cansar.icr.ac.uk/> |
|  | NONCODE | <http://www.noncode.org/> |
|  | ICGC | <https://www.icgc.org/> |
|  | TCGA | <https://www.genome.gov/Funded-Programs-Projects/Cancer-Genome-Atlas> |
|  | CancerResource 2.0 | <http://data-analysis.charite.de/care/> |
|  | ChromoHub V2 | <http://www.thesgc.org/chromohub/> |
|  | DTP | https://dtp.cancer.gov/ |
|  | ICGC Data Portal | <https://dcc.icgc.org/> |
|  | ITTACA | <http://bioinfo.curie.fr/ittaca> |
|  | SCDE | <http://discovery.hsci.harvard.edu/> |
|  | TCGA Roadmap | <http://tcga.github.io/Roadmap/> |
|  | GDC | <https://portal.gdc.cancer.gov/> |
|  | Disease Ontology | https://disease-ontology.org/ |
|  | TSGene | <https://bioinfo.uth.edu/TSGene/> |
|  | Broad Institute | <https://www.broadinstitute.org/cancer/cancer-program-scientific-tools-and-resources> |
|  | NCDB | <https://www.facs.org/Quality-Programs/Cancer/NCDB> |
|  | SEER | <https://seer.cancer.gov/data/> |
|  | COD db | <http://bioinf.modares.ac.ir/software/COD/> |
|  | TIMER2.0 | <http://timer.cistrome.org/> |
|  | GCO | <https://gco.iarc.fr/> |
|  | TCA | <https://canceratlas.cancer.org/> |
|  | OncoDB.HCC | <http://oncodb.hcc.ibms.sinica.edu.tw/index.htm> |
| Expression | CTdatabase | <http://www.cta.lncc.br/> |
|  | dbDEPC 3.0 | <https://www.scbit.org/dbdepc3/index.php> |
|  | DriverDBv3 | http://driverdb.tms.cmu.edu.tw/ |
|  | HPtaa | <http://www.bioinfo.org.cn/hptaa/> |
|  | NCG 4.0 | <http://ncg.kcl.ac.uk/> |
|  | ONCOMINE | <https://www.oncomine.org/resource/login.html> |
|  | OncomiRDB | https://tools4mirs.org/software/mirna_databases/oncomirdb/ |
|  | curatedOvarianData | <http://bcb.dfci.harvard.edu/ovariancancer/> |
|  | MethHC 2.0 | <http://awi.cuhk.edu.cn/~MethHC/methhc_2020/php/index.php> |
|  | miRCancer | <http://mircancer.ecu.edu/> |
|  | RBPTD | <http://www.rbptd.com/#/> |
|  | GEPIA | <http://gepia.cancer-pku.cn/> |
| Gene | IARC TP53 Database | <http://p53.iarc.fr/> |
|  | Mitelman Database | https://mitelmandatabase.isb-cgc.org/ |
|  | RASOnD | <http://www.aiims.edu/RAS.html> |
|  | TGDBs | <http://www.tumor-gene.org/tgdf.html> |
|  | UMD TP53 database | <http://p53.fr/> |
|  | CCDB | <http://crdd.osdd.net/raghava/ccdb/faq.php> |
|  | DDPC | <http://cbrc.kaust.edu.sa/ddpc/index.php> |
|  | G2SBC | <http://www.itb.cnr.it/breastcancer> |
|  | HLungDB | <http://www.megabionet.org/bio/hlung/> |
|  | TSGene 2.0 | <https://bioinfo.uth.edu/TSGene/tutorial.cgi?csrt=3850202211890675783#toc1-1> |
|  | OCDB | <http://www.actrec.gov.in/OCDB/> |
|  | Cancer Genetics Web | <http://www.cancer-genetics.org/X1102.htm> |
|  | IARC TP53 | <https://p53.iarc.fr/> |
|  | SCDb | <http://www.stomachcancerdb.org/> |
|  | HColonDB | <http://diml.ecnu.edu.cn/HColonDB/home/> |
|  | CGC db | <https://cancer.sanger.ac.uk/census> |
|  | PCMdb | <http://crdd.osdd.net/raghava/pcmdb/index.php> |
|  | CGW | <http://www.cancerindex.org/geneweb/> |
|  | HNOCDB | <http://gyanxet.com/hno.html> |
|  | OCG DB | <http://www.actrec.gov.in/OCDB/index.htm> |
|  | CANCROX | <http://cancrox.gmb.bio.br/view/index.php> |
|  | oncoreveal | [https://web.archive.org/web/20110311023352/http://www.oncoreveal.org/](https://web.archive.org/web/20110311023352/http:/www.oncoreveal.org/) |
| Genomic | EGA | <https://www.ebi.ac.uk/ega/> |
|  | GDAC | <http://gdac.broadinstitute.org/> |
|  | canEvolve | [www.canevolve.org/](http://www.canevolve.org/) |
|  | MethyCancer | [http://methycancer.psych.ac.cn](http://methycancer.psych.ac.cn/) |
|  | CGP | <https://www.sanger.ac.uk/group/cancer-genome-project/> |
|  | BioMuta | https://hive.biochemistry.gwu.edu/biomuta |
|  | CGAP | https://mitelmandatabase.isb-cgc.org/about |
|  | TCIA | https://tcia.at/home |
|  | IntOGen | https://www.intogen.org/search |
|  | UCSC Cancer Genomics Browser | <https://genome-cancer.ucsc.edu/> |
|  | Osteosarcoma Database | <http://osteosarcoma-db.uni-muenster.de/> |
|  | YM500 | <http://120.110.158.132:8787/ym500v3/> |
|  | CancerSEA | <http://biocc.hrbmu.edu.cn/CancerSEA/> |
|  | RespCanDB | <http://ridb.subdic-bioinformatics-nitrr.in/> |
| Mutation | COSMIC | [http://cancer.sanger.ac.uk](http://cancer.sanger.ac.uk/) |
|  | SomamiR | <http://compbio.uthsc.edu/SomamiR/> |
|  | CancerDR | <http://crdd.osdd.net/raghava/cancerdr/> |
|  | CGC | <http://cancer.sanger.ac.uk/cancergenome/projects/census/> |
|  | COSMICMart | <https://cancer.sanger.ac.uk/cosmic/login> |
|  | MoKCa | <http://strubiol.icr.ac.uk/extra/mokca/> |
|  | MutaGene | https://www.ncbi.nlm.nih.gov/research/mutagene/ |
|  | COSMIC-3D | <https://cancer.sanger.ac.uk/cosmic3d/> |
|  | CMC db | <https://cancer.sanger.ac.uk/cmc/home> |
|  | Progenetix | <https://progenetix.org/> |
| Proteomic | CPTAC | <https://proteomics.cancer.gov/programs/cptac> |
|  | CanProVar | http://canprovar2.zhang-lab.org/ |
|  | PubMeth | http://www.pubmeth.org/ |
